# “He lets me go although he does not go with me.”: Rwandan women’s perceptions of men’s roles in maternal health

**DOI:** 10.1186/s41256-020-00185-w

**Published:** 2021-01-12

**Authors:** Germaine Tuyisenge, Valorie A. Crooks, Nicole S. Berry

**Affiliations:** 1 Department of Geography, Simon Fraser University, 8888 University Dr, Burnaby, BC V5A 1S6 Canada; 2 Faculty of Health Sciences, Simon Fraser University, Burnaby, Canada

**Keywords:** Maternal health, Gender, Decision-making, Perceptions, Low- and middle- income countries, Rwanda

## Abstract

**Background:**

Increasing men’s involvement in their pregnant partners’ wellness has been reported as one of the ways to improve access to and utilization of maternal health services, including birth preparedness and complication readiness. Men can play meaningful roles in the support systems that pregnant women need to achieve better maternal health outcomes. In Rwanda, the roles that men take vary, resulting in diverse expectations and responsibilities to support the health of women during this critical time. In this study, we aimed to examine the views, perspectives, and experiences of women on men’s involvement in maternal health and how this impacts access and utilization of maternal health services.

**Methods:**

We conducted 21 interviews with pregnant and recently-pregnant women to gain an understanding of their views on men’s involvement in facilitating their partners’ health during pregnancy. Interviews were conducted across five Rwandan districts in both rural and urban settings of the country. Data analysis was guided by a thematic analysis approach. This started with independent transcript review by the investigators, after which a meeting was held to discuss emergent themes and to identify potential codes. A coding scheme was created and transcripts were coded in NVIVO™ software according to conceptual and practical topics that formed an understanding of men’s involvement in maternal care.

**Results:**

Three key themes emerged during the analytic process that categorize the specific roles that men play in maternal health: 1) facilitating access to maternal health services, which involves assisting women with getting and or attending appointments jointly with men; 2) supporting women’s decisions, wherein men can support the decisions women make with regard to their maternal healthcare in a number of ways; and 3) evaluating information, including gathering information from multiple sources, especially from community health workers, to assist women with making informed decisions.

**Conclusion:**

Rwandan men take on three types of roles in supporting women’s maternal health, and their responsibilities are experienced differently by women. Interventions involving men are encouraged to increase their understanding of the implications of their involvement in maternal health without compromising women’s autonomy in decision-making and to promote positive maternal health outcomes.

## Background

Poor maternal health outcomes (e.g., maternal mortality and morbidity) continue to be documented in countries around the world, and are most significant in low- and middle-income countries [1–3]. In recent decades, several initiatives have been implemented within countries and regions to promote improved maternal outcomes by reducing maternal mortality and promoting access to reproductive health [1, 2, 4]. Some of these initiatives include: promoting antenatal care and access to skilled birth attendance, encouraging the uptake of family planning methods, and increasing the number of trained maternal healthcare professionals [5–8]. Increasing community engagement in maternal healthcare has been another widely-adopted strategy, where community members are actively involved in the implementation and oversight of community-based initiatives that serve to promote better maternal health experiences [9, 10]. Despite such initiatives, however, little-to-no progress in ending preventable deaths of women has been observed among countries that reported the highest maternal mortality ratio. This is due to poor access to affordable interventions and maternal health inequities that are still observed in these countries [11–13]. As such, there is a great need to look at varying ways to involve an even wider range of actors in new initiatives, including men’s active involvement at different stages of pregnancy to promote positive maternal health outcomes [14, 15].

Men’s involvement in maternal health as partners to pregnant women is an area that has received growing attention in recent years as significant to promoting better maternal health outcomes [16, 17]. Men’s involvement with their partners’ pregnancies typically centres around building knowledge of maternal health, being part of or supporting informed decision-making, attending appointments, and caring for pregnant partners [18, 19]. The World Health Organization (WHO) states that men’s involvement in maternal health is vital to support improvements in maternal health outcomes [20]. The WHO further advises that men’s involvement in improving maternal health outcomes can be carried out without negatively compromising women’s autonomy [20]. The WHO’s recommendations suggest having men involved at all stages of maternal care, from pregnancy through to delivery, as well as building a local culture of men’s participation in maternal health. This calls for both partners, where appropriate, to be fully engaged in the pregnancy and delivery processes and to have specific plans to ensure that women receive timely care [21].

In patriarchal cultures, maternal health is frequently thought of as primarily a women’s issue which may result in limiting the scope of men’s involvement [15, 16, 22]. For example, a review of what determines men’s involvement in maternal and child health services in sub-Saharan Africa [23] highlighted that a partner’s pregnancy tended to produce very limited societal expectations of men, with involvement being limited to activities such as financial support and/or accompanying their partners for required antenatal testing of the human immunodeficiency virus and acquired immunodeficiency syndrome (HIV/AIDS). This review found that such participation was influenced by socio-demographic and sociological factors, including education, income, and cultural beliefs, as well as factors related to the availability of services and the accommodation of male partners [23].

The African nation of Rwanda has made remarkable efforts to improve maternal health during the last decade. Most of these efforts are aligned with the country’s progress towards promoting gender equity [8, 24]. Maternal health initiatives have been implemented alongside other socio-economic development initiatives during the rebuilding process of the country following the 1994 genocide against the Tutsis [8, 25]. Such initiatives include, for example, the education of girls, the increasing roles of women in key decision-making institutions, and the fight against the HIV/AIDS epidemic [25]. Not only there has been noticeable government will for promoting maternal health as part of the country’s larger rebuilding efforts, but Rwandan communities have also come to recognize the importance of ensuring that women experience positive maternal health by receiving local support [26, 27]. Despite these collective efforts, little is known about the role that male partners play in supporting and promoting maternal health in Rwanda. The current analysis seeks to address this knowledge gap through qualitatively exploring Rwandan women’s perspectives on men’s involvement in maternal care. Specifically, we ask: what are women’s experiences and expectations of men’s involvement in maternal health as their partners, and how does their involvement affect overall access to health services during pregnancy?

In Rwanda, it is expected that male partners will accompany pregnant women for the first antenatal care appointment so that both partners can be tested for HIV/AIDS [28]. Both partners are tested so that the mother can be given appropriate treatment to protect mother-to-child transmission in instances where either partner has tested HIV+ [8]. This is the only form of men’s involvement in maternal care that is formally required by local health centres. Beyond this requirement, the initiative offers no guidelines to advise on potential roles for men in promoting maternal health or caring for pregnant partners. It has been suggested elsewhere that the patriarchal nature of Rwandan society leads to expectations that men make key household decisions on their own, and in some instances men’s decision-making extends into the realm of maternal care, such as by choosing where and when care will be accessed [29]. Existing research on maternal care in Rwanda has not fully explored men’s involvement in this form of care from women’s perspectives, nor considered the expectations that women have regarding the involvement of their partners during this period. In this study, we aimed to examine the views, perspectives, and experiences of women on men’s involvement in maternal health and how this impacts access and utilization of maternal health services. Here we report on the thematic findings of interviews conducted with 21 pregnant or recently-pregnant Rwandan women as a first step towards exploring these perspectives and expectations. These insights can assist with informing the development of maternal health interventions that meaningfully incorporate men’s involvement.

## Methods

### Design

This exploratory analysis contributes to a larger case study that included interviews with both voluntary community maternal health workers and women who have used their services in order to explore Rwandan women’s access to maternal healthcare at the community level. In this analysis we draw solely from the in-depth interviews conducted with pregnant women and recently-pregnant women (i.e., new mothers) in both rural and urban settings. These interviews were conducted in June and July of 2017.

### Settings

Following ethics approvals from both Simon Fraser University’s Research Ethics Board and Rwanda’s Ministry of Education, we conducted interviews with women living in five districts of Rwanda. The districts are: Nyarugenge and Gasabo in the Kigali City Province (urban), Gakenke and Rulindo in the Northern province (rural), and Ruhango in the Southern Province (rural). In each district, women accessing care from one of two participating community health centres were invited to participate. The following health centres participated: Bumbogo and Gatsata in Gasabo district; Gitega and Kimisagara in Nyarugenge district; Ruli and Nemba in Gakenke; Shyorongi and Kinihira in Rulindo; and Byimana and Kinazi in Ruhango. A larger international project (the Training, Support and Access Model for Maternal, Newborn and Child Health project) focused on devising and evaluating maternal health interventions assisted with identifying health centres willing to assist with recruitment for the current study. We further consulted with local authorities and health directors in each district in order to confirm the most suitable health centres to assist with participant recruitment.

The urban and rural nature of the communities in which data collection took place had similarities and differences in socio-cultural and economic contexts. Culturally, men in Rwanda are considered the breadwinners and dominant decision-makers in health-related matters, including maternal health [30]. Also, there are no socio-cultural expectations for men to be involved in providing care for infants and children. However, these entrenched gender roles are being reformed, which is influenced by more women becoming educated and or participating in income-generating activities [31]. These changes are informing a broader social desire to achieve gender equality in the country in both urban and rural communities, including as this relates to maternal health. An important difference between the rural and urban communities of focus related to infrastructure. Access to healthcare is limited in rural Rwanda [32]. In addition, rural communities typically have limited access to transportation and communication infrastructure. Meanwhile, urban communities have paved roads, reliable electricity, and local healthcare clinics.

### Recruitment

We sought to interview women living in the five districts of focus who were pregnant or had given birth within the previous year. We aimed to speak with women who had lived in their current village for at least a year, and who had accessed maternal care at the village level. Participants were recruited with the help of the coordinators of community health workers in each of the health centres, who provided written and verbal information about the study to potential participants. About thirty potential participants received information. Ultimately, 21 women contacted the interviewer (GT) to gain more information about the study and schedule an interview. Recruitment and data collection were guided by a temporal cut-off, which was the point at which this fieldwork was scheduled to end. The interviewer, who is also Rwandan and who is fluent in Kinyarwanda (Rwanda’s national language), arranged to meet women in-person at their homes or in a location of their choosing. To maintain confidentiality, the community health worker coordinators were not informed of who ended up participating in the study. In order to compensate participants for their time and express appreciation for their contribution to the study, a small token (a package of rice or sugar) was given as a gift.

### Data collection

A semi-structured questionnaire was used to guide the in-person interviews. Open-ended questions were asked to elicit views from women on their understanding of men’s involvement in maternal health and factors that affected women’s access to health services during pregnancy and after childbirth. Such questions provided a general overview on access to maternal health services in the community, including understanding the appointment day and any related household and community dynamics regarding attending appointments. Results on these factors have been published elsewhere [31, 33] as part of a larger study that qualitatively explored the aspects of access to community-based maternal health services in Rwanda.

Verbal consent was obtained from each participant; they were assured of confidentiality, anonymity, and freedom to withdraw from the study at any stage. The beginning of each interview was marked by participants providing socio-demographic information to help the research team characterize the participant group. Interviews lasted between 45 to 70 min, were conducted in Kinyarwanda, and were audio-recorded with participants’ permission. Body language, as well as field notes, were also recorded and incorporated alongside interview transcripts. The researcher prearranged the locations with participants before the interview.

### Data analysis

Interviews were transcribed and then translated verbatim from Kinyarwanda to English alongside the field notes. A thematic analysis approach guided data analysis. This started with the investigators’ independent transcript review, after which a meeting was held to discuss emergent themes and identify potential codes.. A coding scheme was created and transcripts were coded in NVIVO™ software according to conceptual and practical topics that formed an understanding of men’s involvement in maternal care. Coding extracts were reviewed by team members to confirm the scope and scale of the emerging themes. Consistent with thematic analysis, the themes were also contrasted against existing knowledge of the topic to identify novel contributions. In the following section, we share the analytic findings, incorporating direct quotes to assist with facilitating analytic trustworthiness.

## Results

We conducted a total of 21 interviews with women between 19 and 39 years of age. For the most part, participants were married and lived with a partner. There were a few single mothers who lived either on their own or with their parents. Two participants were pregnant for the first time, and the rest had children already, aged between 6 months and 12 years old. All participants had completed 6 years of primary education, and a few had started additional years of secondary education, although none had completed this level of education. In terms of economic activities, participants were mostly farmers, while a small number were either wage-earning workers, tradespeople, or stay-at-home mothers.

The following section highlights the three key themes that categorize the specific roles that men have in maternal health that emerged during the analytic process. The first theme, facilitating access, relates to ways by which men can enable women to get to appointments, for example, by taking on additional household duties on appointment days, providing funds for transportation, and attending appointments. The next theme, supporting decisions, addresses ways men can support women’s informed decisions about maternal care and in taking lead in decision-making. Lastly, the theme of evaluating information pertains to how men can take in information from other sources, especially from community health workers, to assist them in understanding their roles and responsibilities. Where applicable, we use participants’ direct quotes to enable their voices to illustrate the themes that emerged from the analysis.

### Facilitating access

Participants shared different ways by which their partners supported them in accessing maternal healthcare services. For example, one participant shared that her partner helped with household tasks on appointment days, which made it easier for her to attend: “*It makes me feel good to see my husband helping me at home … I do not ask him to do that … that is something most men do not do*.” Other participants shared that male partners enabled women to go to appointments by taking on additional farming duties on appointment days. In this context, one participant shared the following: “*He lets me go even though he does not go with me. Some men forbid their wives to go; they do not want them to lose the hours of farming because they do not think antenatal care is that important*.” For other participants, they talked about how their partners facilitated access by providing funds related to attending maternal health services. Financial access was facilitated in different ways. As one participant shared: “*I ask my husband if he has money to give me for lunch [on appointment days] … I have to wait until he has money … he keeps that in mind though … and makes sure I go as soon as possible*.” Another participant reiterated the former, saying: “*My husband decides [when appointments get scheduled]. He told me to wait for this week to end so that he can be paid and be able to buy us [with the baby] new clothes before we can come*.” Financial support played a vital role in facilitating access and was often related to the decision-making regarding when to use maternal health services.

A few participants revealed that their partners provided support through attending appointments with them: “*When I tell my husband that I want to go to the health centre, he understands it well … Or that I do not feel good and want him to go with me to the health centre, he accepts*.” In terms of attending appointments, participants shared that their male partners typically only attended the first antenatal care appointment together, as it was a requirement from the Ministry of Health that both partners get tested for HIV/AIDS to prevent mother-to-child transmission. Participants explained that they had to find a time that worked for both partners to attend the appointment together. A participant mentioned this: “*We have to see when will be a most convenient day for him … because that has to depend on what kind of work he has and where … he is in construction, and they move from one place to another*.” In this context, the partner’s availability, in addition to the willingness to go, was crucial to ensuring the expectant mother received maternal health services on time.

### Supporting decisions

Participants highlighted that in some cases their male partners supported the decisions that they and other women made concerning their health and maternal care by acknowledging choices, affirming decisions, and facilitating women’s leadership in decision-making. For example, one participant mentioned: “*I make the decision … when I have an appointment, I try my best to remember. I would ask him what day we are on and tell him when I have an appointment. I do not have to tell him twice*.” Some participants stressed that they were the primary decision-makers for maternal health given the implications for their health, wellbeing, and bodies. In this context, a participant mentioned that: “*You have to be the first to make the decision [to access care] because, for instance, when you have a health concern, you are the first one to notice and you make the decision to go to the health centre*.” This was emphasized by another participant who said: “*Of course your spouse supports you, but you are the first to make the decision.*” These examples showed that men were involved in different ways in supporting decisions related to maternal health. The examples also showed that men’s roles were negotiated and that some women took charge of their health and also in deciding to what extent their male partners would be involved.

There were cases where men were not involved and where women were the sole decision-makers on matters of maternal health. This was mostly when it came to navigating aspects of roles that were heavily socially and culturally gendered. For example, one participant shared that: “*I make the decisions myself because men are not good at maternal health things. Like from the time I gave birth until now, my husband does not even know that I take the baby for vaccination*.” This participant continued by explaining the reason behind her decision-making when it came to maternal health: “*I am the one that has to do it … I have to make decisions.*” As shown by these quotes, in some cases, decision-making extended almost seamlessly from maternal health to child health. In some instances, women felt more knowledgeable about maternal and child health and did not believe their male partners needed to contribute to key decision-making. In such cases, men’s support may have seemed to be lacking because their opportunities for participation were minimal.

### Evaluating information

Many participants explained that a meaningful opportunity for their male partners to participate would be to help accessing and coordinating information from various sources, especially from maternal community health workers (M-CHWs). Through becoming familiar with information on maternal health, some participants indicated that this was a way for men to gain insight into their potential roles and responsibilities. This was mostly highlighted by participants who shared that they preferred to receive maternal health information in the presence of their partners so that they were both on the same page when it came to birth preparedness. One participant explained: “*It is helpful when community health workers come and give information in the presence of the spouse … that helps him to understand more about household chores and nutrition … because that makes him help me*.” When asked why this was important to her, this participant commented: “*It might be different if I just tell him what the community health worker said by myself … maybe he would not believe me.*” The same sentiment was shared by another participant who said that: “*Community health workers help convince our partners to help us for preparations. We used to ask them [male partners] for money to buy baby clothes, and they would say that one cannot buy clothes for a child that is not born yet*.” When asked about the difference that it made to have men involved in education on maternal health, participants stressed that it helped in birth preparedness.

For some women, dynamics related to maternal health strained their relationships with their partners. Such strain on the relationship affected men’s participation in maternal health. This was the case, for example, for unintended pregnancy, as shared by a participant who said: “*Our relationship got worse when I told him that I was pregnant; he got mad at me and refused even to accompany me to the health centre.*” In such cases, the local authorities, including M-CHWs, may have had to intervened to encourage a man’s participation in maternal health through providing information and opening the opportunity for their involvement. As one participant explained: “*Community health workers supported me...to even go to the health centre, local authorities had to intervene … they wanted to talk to him, but he hid from them, and I do not know where he lives*.” This example serves as a reminder that a number of actors may be involved in supporting men’s involvement in maternal health and providing them with integral information beyond the expectant mother and her family.

## Discussion

This study highlights different aspects of Rwandan men’s roles in maternal health from women’s perspectives, including their roles in facilitating access, supporting decisions, and evaluating information. The current analysis stresses the relationship between men’s participation and increased access to maternal health services. As highlighted by participants of this study, men’s roles in maternal health impact the financial, physical, and social aspects of women’s access to maternal healthcare services. For example, men’s involvement in maternal health increases the use of sexual and reproductive health services and self-care for women during pregnancy. In terms of supporting decisions, this study points out the roles that Rwandan men can and do play in their partners’ decision-making regarding maternal health, including supporting women’s autonomy around the use of maternal health services. Finally, men’s abilities to evaluate information relate to the ways in which community members, including M-CHWs, play a crucial role in getting men informed about maternal health along with their partners. Information from various sources can inform men about their responsibilities to their pregnant partners and what is expected from them in this critical period of women’s health.

This study confirms the results of other African studies that show that for some, men’s involvement in maternal health is limited to becoming active in emergency situations, which can delay the decision to utilize maternal health services due to lack of knowledge about the spectrum of maternal care [22, 34]. For example, a study in Tanzania that applied men’s involvement to the “three delays model” emphasized the impact that men’s involvement in maternal health can play in the utilization of healthcare. This model consists of men being informed about when and where to seek care, financial support for their partners, and satisfaction/dissatisfaction with services provided to their partners [19]. Another study conducted in Ghana shows that men were more involved in their partner’s maternal health in case of pregnancy or birth complications [35], hence suggesting little-to-no support for non-emergency maternal health situations. Similarly, the results of a study in Uganda found that through the traditional roles of men and women, men regarded maternal health as solely women’s business, and that men’s involvement in maternal health was to be restricted to providing financial support [36]. The fact that other studies conducted in nearby countries that have some cultural continuity with Rwanda also point to men’s limited involvement in maternal health may suggest that a *regional* approach to strengthening men’s participation in such care may be useful.

The participants in this study offered two different perspectives on how they understood Rwandan men’s roles in maternal health. On one hand, some women wanted to have their partners involved in more aspects of maternal health, such as having their physical presence when they attend maternal health services, both antenatal care and labour – which is a view documented elsewhere [37]. On the other hand, some women wanted their male partners to undertake discrete tasks or have specific forms of involvement in maternal health, such as attending appointments – a perspective that has also been shown in other studies [37]. Meanwhile, many participants indicated that it was common for Rwandan men to have almost no involvement in maternal health and focusing solely on providing money to cover the costs of women’s care and care for the newborn. Undertaking activities such as attending appointments is sometimes viewed as a financial loss as time is taken off of work and there may be food and transportation costs incurred, and while this is viewed as a justifiable expense for one partner it may be viewed as a burdensome expense when both partners attend. A significant socio-cultural shift is needed in order to support Rwandan men in viewing their roles in maternal health, including through attending appointments, to be an investment and not a burden and even to find ways to lessen financial barriers to men’s involvement in maternal health.

The current analysis shows gaps in understanding the scope of men’s roles in maternal health. This gap is echoed in other studies in sub-Saharan Africa, including a study conducted in Nigeria that showed that men do not always know what is expected of them [38], and expectations can vary by community, family and individual. And for some communities (e.g Northern Ghana), women deliberately refuse to be accompanied by their male partners to pregnancy-related appointments because it is perceived as form of public display of affection and is considered inappropriate [39]. This uncertainty not only hinders the involvement of men in maternal health, but it also may construct a barrier to the support they need regarding some aspects of maternal health such as adverse pregnancy outcomes [40, 41]. These other studies help to underscore the reality that men’s full integration into maternal health in Rwanda will not be possible until social attitudes such as these are shifted in a way that does not stigmatize their participation. Subsequent research with Rwandan men is greatly needed in order to identify if and how stigma and other cultural dimensions play a role in shaping their attitudes towards participating in maternal health.

In Sub-Saharan Africa, several strategies have been used to promote men’s involvement in maternal health. A qualitative study conducted in Rwanda [30] showed that there are men who would like to be more involved in their partners’ maternal health, following the country’s efforts to promote gender equality [26]. Some men want to go beyond the traditional roles assigned to them of being financial providers and companions to the first antenatal care visit only [30]. In this regard, studies elsewhere [42, 43] showed that having the support of religious leaders, local leaders, and community health workers is crucial to increasing male involvement in maternal health. For example, in Malawi, strategies to increase men’s participation in maternal health are implemented at different levels, including in the community and at the point of health provision [44]. The community-level involves mobilizing families and communities by using peers and the provision of incentives for men who are involved in maternal health. The health facility level consists of involving men in maternal health when women need treatment for sexually transmitted infections, including HIV, in order to treat her partner and prevent transmission to the baby [44]. Initiatives such as these show promise regarding improving maternal health outcomes as well. Given that this is a goal in Rwanda, undertaking similar interventions to support men’s involvement in maternal health may lead to improving maternal health outcomes and decreasing maternal mortality overall.

There are limitations to this study, and here we identify two that are most significant. First, interviews were conducted in Kinyarwanda and then translated into English in the process of verbatim transcription. It is likely that some conversational nuance was lost in the process of the translation. We also did not have the means to back-translate quotes selected for inclusion in this article back into Kinyarwanda to confirm the integrity of the interpretation. However, the first author is fluent in Kinyarwanda and so her involvement assists with mitigating this limitation. Second, we used a temporal cut-off for data collection instead of being guided by other approaches, such as data saturation or theoretical saturation. A limitation of using a temporal cut-off was that we had to complete data collection at a certain point of time regardless of how robust the dataset was. As there are limitations associated with all aspects of data collection, we do not believe those associated with using a temporal cut-off for data collection are any more significant than those associated with other methods.

## Conclusion

This study has emphasized that Rwandan men’s involvement in different roles related to maternal health, and their responsibilities for specific tasks are understood in particular ways by women. Interestingly, it is also not clear whose role it is to promote men’s involvement in maternal health: women themselves, the Ministry of Health, community health centres, or others. Previous research on promoting maternal health in Rwanda highlights the role that community health workers play as key stakeholders in linking communities and their members to the formal healthcare system. They are thus a logical group to involve in strategies to promote men’s involvement in maternal health. Such strategies must focus on identifying couples’ needs and challenges to men’s involvement in maternal health and find appropriate, culturally-sensitive solutions to them. In order to increase positive maternal health outcomes in Rwanda, community-level discussions involving men and their roles in maternal healthcare are encouraged. Such discussions will increase men’s understanding of the implications of their involvement in maternal health without compromising women’s autonomy in decision-making on maternal health.

## Data Availability

The datasets generated and analyzed during the current study are not publicly available due to the respondents’ consent to use the data for this research specifically. Data can be available upon reasonable request to the corresponding author.

## Abbreviations

M-CHW: Maternal Community Health Worker
HIV: Human Immunodeficiency Virus
AIDS: Acquired Immune Deficiency Syndrome
